# Hypertension is associated with oral, laryngeal, and esophageal cancer: a nationwide population-based study

**DOI:** 10.1038/s41598-020-67329-3

**Published:** 2020-06-24

**Authors:** Jae-Hyun Seo, Young-Du Kim, Chan-Seok Park, Kyung-do Han, Young-Hoon Joo

**Affiliations:** 1 Department of Otolaryngology-Head and Neck Surgery, College of Medicine, The Catholic University of Korea, Seoul, Korea; 2 Department of Thoracic Surgery, College of Medicine, The Catholic University of Korea, Seoul, Korea; 3 Division of Cardiology, Department of Internal Medicine, College of Medicine, The Catholic University of Korea, Seoul, Korea; 4 Department of Biostatistics, College of Medicine, The Catholic University of Korea, Seoul, Korea; 5 Cell Death Disease Research Center, College of Medicine, The Catholic University of Korea, Seoul, Korea; 6 Department of Otolaryngology, Head and Neck Surgery, Bucheon St. Mary’s Hospital, College of Medicine, The Catholic University of Korea, 2 Sosa-dong, Wonmi-gu, Bucheon, 420-717 Kyounggi-do Korea

**Keywords:** Cancer epidemiology, Risk factors

## Abstract

Several studies have reported an association between hypertension and upper aerodigestive tract cancer, but no large-scale, population-based studies have been conducted to confirm this.The aim of this study was to explore the association between hypertension and risk of upper aerodigestive tract cancer in Koreans. Participants who underwent a national health screening examination from January 1 to December 31, 2009 (n = 9,746,606) were enrolled. We assessed the development of oral, laryngeal, or esophageal cancer until 2016 using records from the Korean Health Insurance claims database during the study period. During the seven-year follow-up period, 6,062, 2,658, and 4,752 subjects were newly diagnosed with oral, laryngeal, and esophageal cancer, respectively. Participants with metabolic syndrome had the highest risk of developing oral cancer (hazard ratio (HR) 1.09, 95% confidence interval (CI) 1.03–1.16), laryngeal cancer (HR 1.27, 95% CI 1.17–1.38), and esophageal cancer (HR 1.11, 95% CI 1.04–1.19). Hypertension was a remarkable risk factor for each cancer (HR 1.11, 95% CI 1.04–1.17 for oral cancer; HR 1.23, 95% CI 1.13–1.33 for laryngeal cancer; HR 1.25, 95% CI 1.18–1.33 for esophageal cancer) after adjusting for age and other variables including gender, smoking status, alcohol intake, exercise, body mass index, and diabetes. Patients with untreated hypertension were at highest risk of developing oral cancer (HR 1.15; 95% CI 1.05–1.26), laryngeal cancer (HR 1.25; 95% CI 1.09–1.44), and esophageal cancer (HR 1.47; 95% CI 1.33–1.63) after adjusting for confounders. Hypertension was associated with the risk of oral, laryngeal, and esophageal cancer, despite of the lack of detailed biochemical information including the cancer cell types (squamous cell carcinoma or adenocarcinoma), cancer stage, physical findings and other medical history. Further studies are warranted to determine the reasons for this association and to establish effective interventions in this vulnerable population.

## Introduction

One million new cases of upper aerodigestive tract (UADT) cancers are diagnosed worldwide every year, and they are ranked among the top ten most common cancers. Malignant tumors of the oral cavity, oropharynx, larynx, and esophagus comprise almost (or more than) 90% of all cancers of the UADT^1^. Most of these cancers are histologically squamous cell carcinoma. Although the UADT is not considered a vital organ, it is closely associated with quality of life as it relates to eating, breathing, and speaking. Malignancies of this organ are therefore highly morbid. As with all other malignant tumors, identifying and minimizing risk factors for UADT cancer development is essential. Alcohol consumption and tobacco smoking are well known risk factors for these cancers^1,2^.

Metabolic syndrome (MetS) is a cluster of metabolic abnormalities associated with insulin resistance, including obesity, hypertension, hyperglycemia, dyslipidemia, and elevated triglyceride levels^3^. MetS is closely linked to cancer, as it increases cancer risk and cancer-related mortality^3^. Early diagnosis of MetS is crucial in those with malignant tumors. The incidence of MetS is gradually increasing in Korean adults. Since there are reports that MetS is correlated with the occurrence of malignant tumors, we must verify the relationship between MetS and cancer development through population studies^4^.

Hypertension is the most prevalent adult disease in South Korea, affecting 7.8% of Korean adults^5^. In 2017, US guidelines for hypertension changed the definition of hypertension from the general accepted level of 140/90 mmHg to 130/80 mmHg ^6^. However, the recently announced Korean hypertension guidelines maintained 140/90 mmHg as the definition criteria of hypertension^7^. Many studies have separately reported hypertension as an important risk factor for rising cancer incidence and mortality. Hypertension is an independent risk factor of renal cell carcinoma based on prospective cohort study^8^. In addition, the results from other large prospective studies reported positive associations of hypertension with the risk of cancers in locations other than the kidney in men (oropharynx, colon, rectum and anus, lung with larynx and trachea, bladder, malignant melanoma and non‐melanoma skin cancer)^9^. The meta-analysis of four prospective studies between hypertension and esophageal adenocarcinoma yielded a statistically significant positive association^10^. Most recently, Christakoudi et al. reported that there was a positive correlation between blood pressure and risk of esophageal carcinoma and head and neck cancers in a prospective European study^11^. However, there have been no large-scale, population-based studies investigating whether hypertension increases the risk of developing UADT cancers. The objective of this study was to determine the effect of hypertension on the development of oral, laryngeal, and esophageal cancer in a Korean population.

## Results

### Basic characteristics

Baseline characteristics of the population are summarized in Table 1. The mean age of those with hypertension was significantly higher than that of those without hypertension (56.57 years vs. 43.59 years, p < 0.0001). The baseline percentage of male participants with hypertension was 56.96% and the percentage of those without hypertension was 54.26% (p < 0.0001). Current smoking, heavy drinking, diabetes, and dyslipidemia were significantly more frequent in the hypertension groups at baseline. Participants with hypertension had significantly higher body mass index, mean waist circumferences, systolic blood pressure (SBP), diastolic blood pressure (DBP), fasting glucose levels, and fasting total cholesterol levels than those without hypertension (p < 0.0001).

**Table 1:** Analysis of factors potentially associated with hypertension (n = 9,746,606).

Parameter	Yes (n = 2,481,444)	No (n = 7,265,162)	P-value
Age (years)	56.57 ± 12.80	43.59 ± 12.87	< 0.0001*
Gender (male)	1,413,350 (56.96%)	3,942,240 (54.26%)	< 0.0001*
Smoking (current smoker)	2,022,811 (27.84%)	555,105 (22.37%)	< 0.0001*
Drinking (heavy drinker)	203,531 (8.2%)	470,834 (6.48%)	< 0.0001*
Routine exercise	1,210,766 (48.79%)	3,805,517 (52.38%)	< 0.0001*
Place (urban)	1,349,490 (54.38%)	3,913,772 (53.87%)	< 0.0001*
Diabetes	477,770 (19.25%)	357,521 (4.92%)	< 0.0001*
Dyslipidemia	820,382 (33.06%)	951,895 (13.1%)	< 0.0001*
Body mass index (kg/m^2^)	25.03 ± 3.22	23.26 ± 3.07	< 0.0001*
Waist circumference (cm)	84.56 ± 8.45	78.73 ± 8.80	< 0.0001*
Systolic BP (mmHg)	136.34 ± 15.58	117.62 ± 11.26	< 0.0001*
Diastolic BP (mmHg)	84.54 ± 10.76	73.48 ± 7.92	< 0.0001*
Glucose (mmol/L)	5.82 ± 1.61	5.24 ± 1.09	< 0.0001*
Total cholesterol (mmol/L)	5.17 ± 1.00	5.00 ± 0.93	< 0.0001*

Values are mean ± SE or % ± SE.

*Significant at p < 0.05.

### Association of MetS with oral, laryngeal, and esophageal cancers

During the seven-year follow-up period, 6,062, 2,658, and 4,752 subjects were newly diagnosed with oral, laryngeal, and esophageal cancers, respectively. We analyzed the effect of MetS at baseline on the risk of each cancer (Table 2). Age, gender, smoking status, alcohol intake, exercise, and body mass index-adjusted hazard ratios indicate that participants with MetS had a higher risk of developing each cancer [hazard ratio (HR) 1.09, 95% confidence intervals (CI) 1.03–1.16 for oral cancer, HR 1.27, 95% CI 1.17–1.38 for laryngeal cancer, and HR 1.11, 95% CI 1.04–1.19 for esophageal cancer) than did those without MetS. The number of MetS components was found to be a strong risk factor, with a higher risk estimate of oral, laryngeal, and esophageal cancers.

**Table 2:** Multivariable Cox proportional hazard model for incidence of oral, laryngeal, and esophageal cancer according to the metabolic syndrome.

Variables	Total Number	Oral cancer				Laryngeal cancer				Esophageal cancer			
		No of cases	Person-years	Annual incidence rates	Hazard ratio (95% CI)	No of cases	Person-years	Annual incidence rates	Hazard ratio (95% CI)	No of cases	Person-years	Annual incidence rates	Hazard ratio (95% CI)
Metabolic syndrome													
No	7,150,403	3,822	52,098,106	0.07336	1 (reference)	1,609	52,104,839	0.03088	1 (reference)	3,044	52,102,807	0.058423	1 (reference)
Yes	2,596,042	2,240	18,778,185	0.11929	1.09 (1.03–1.16)	1,049	18,781,282	0.055853	1.27 (1.17–1.38)	1708	18,780,664	0.090945	1.11 (1.04–1.19)
Number of metabolic syndrome													
0	2,521,362	911	18,440,641	0.0494	1 (reference)	303	18,442,567	0.015429	1 (reference)	506	18,442,286	0.02744	1 (reference)
1	2,576,107	1,421	18,761,529	0.07574	1.04 (0.95–1.13)	607	18,763,978	0.032349	1.14 (0.99–1.31)	1,247	18,763,004	0.06646	1.39 (1.25–1.54)
2	2,052,934	1,490	14,895,935	0.10003	1.12 (1.03–1.22)	699	14,898,293	0.046918	1.35 (1.17–1.55)	1,291	14,894,516	0.08666	1.49 (1.34–1.66)
3	1,417,283	1,159	10,263,774	0.11292	1.14 (1.04–1.26)	558	10,265,329	0.054358	1.48 (1.27–1.71)	888	10,265,042	0.08651	1.43 (1.27–1.60)
4	843,804	771	6,098,914	0.12642	1.19 (1.07–1.32)	367	6,099,957	0.060164	1.61 (1.37–1.90)	613	6,099,724	0.1005	1.65 (1.46–1.87)
5	334,955	310	2,415,497	0.12834	1.22 (1.06–1.41)	124	2,415,995	0.051325	1.65 (1.32–2.07)	207	2,415,897	0.08568	1.72 (1.44–2.04)

*Model* Adjusted for age, gender, smoking status, alcohol intake, exercise, and body mass index.

### Association of hypertension with oral, laryngeal, and esophageal cancers

**Figure 1:**
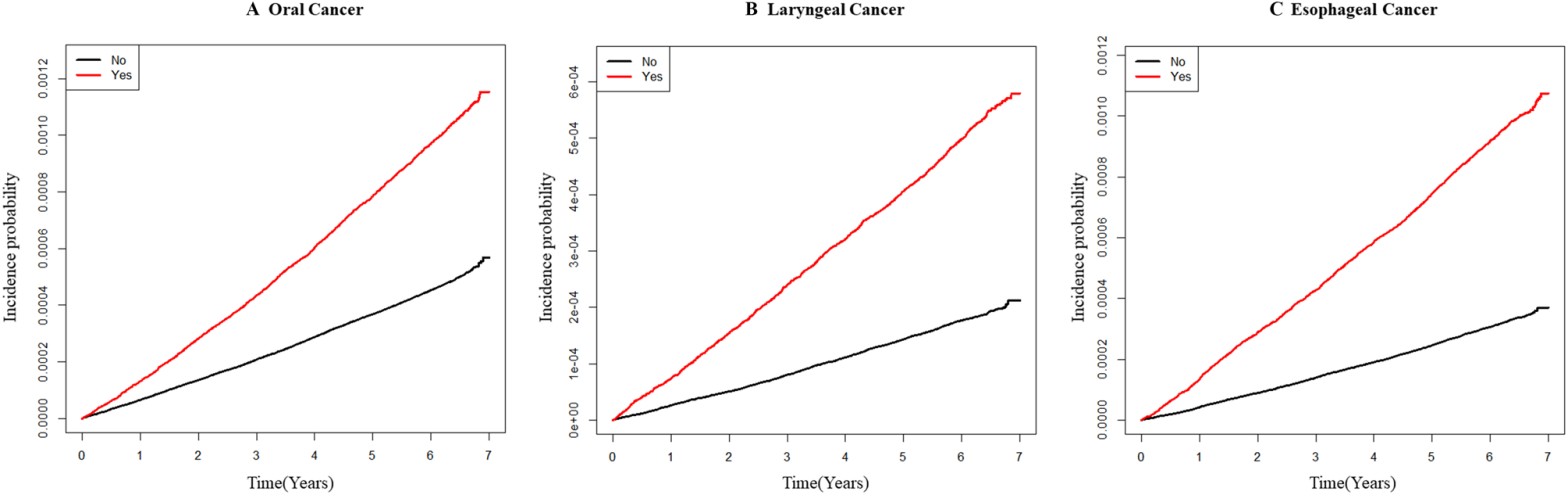
The risk of cancer according to the presence of hypertension. (**A**) Oral cancer, (**B**) Laryngeal cancer, (**C**) Esophageal cancer. The X-axis represents years, while the Y-axis represents the cumulative incidence probability of cancer occurrence.

The relationship between hypertension and the risk of oral, laryngeal and esophageal cancers was linear as shown in Figure 1. Hypertension was significantly associated with an increased risk of each cancer. Table 3 shows results of Cox proportional hazards analyses after adjusting for age and other variables including gender, smoking status, alcohol intake, exercise, body mass index, and diabetes for each cancer. Hypertension was a notable risk factor for each cancer after adjusting for confounders (HR 1.11, 95% CI 1.04–1.17 for oral cancer; HR 1.23, 95% CI 1.13–1.33 for laryngeal cancer; HR 1.25, 95% CI 1.18–1.33 for esophageal cancer). When participants were divided according to gender or smoking status, the risk of developing each cancer was significantly associated with hypertension in men (HR 1.14, 95% CI 1.06–1.21 for oral cancer, HR 1.24. 95% CI 1.14–1.35 for laryngeal cancer, and HR 1.30, 95% CI 1.22–1.38 for esophageal cancer) and smokers (HR 1.22, 95% CI 1.11–1.34 for oral cancer, HR 1.34, 95% CI 1.20–1.50 for esophageal cancer, and HR 1.46, 95% CI 1.33–1.60 for esophageal cancer). We also estimated the risk of incident cancer in those with hypertension according to smoking and alcohol consumption. Among even non-drinkers and non-smokers, the HR for hypertension was 1.17 (95% CI 1.07–1.28) for esophageal cancer.

**Table 3:** Multivariable Cox proportional hazard model for incidence of oral, laryngeal, and esophageal cancer according to the presence or absence of hypertension.

Variables	Hypertension	Oral cancer				Laryngeal cancer				Esophageal cancer			
		No of cases	Person-years	Annual incidence rates	Hazard ratio (95% CI)	No of cases	Person-years	Annual incidence rates	Hazard ratio (95% CI)	No of cases	Person-years	Annual incidence rates	Hazard ratio (95% CI)
All	Yes	2,535	1,55,07,322	0.16347	1.11 (1.04–1.17)	1,301	1,55,09,717	0.083883	1.23 (1.13–1.33)	2,378	1,55,08,598	0.15333	1.25 (1.18–1.33)
	No	3,527	4,58,93,482	0.07685	1 (reference)	1,357	4,58,98,615	0.029565	1 (reference)	2,374	4,58,97,503	0.05172	1 (reference)
Male	Yes	1,932	87,66,081	0.22039	1.14 (1.06–1.21)	1,239	87,67,241	0.14132	1.24 (1.14–1.35)	2,206	87,66,266	0.25165	1.30 (1.22–1.38)
	No	2,571	2,48,40,630	0.1035	1 (reference)	1,272	2,48,43,442	0.0512	1 (reference)	2,129	2,48,42,595	0.0857	1 (reference)
Female	Yes	603	67,41,241	0.089449	1.05 (0.93–1.18)	62	67,42,476	0.009195	1.00 (0.69–1.45)	172	67,42,332	0.02551	0.90 (0.72–1.11)
	No	956	2,10,52,851	0.04541	1 (reference)	85	2,10,55,172	0.004037	1 (reference)	245	2,10,54,907	0.01164	1 (reference)
Smoker	Yes	820	34,32,921	0.23886	1.22 (1.11–1.34)	641	34,33,138	0.18671	1.34 (1.20–1.50)	967	34,33,003	0.28168	1.46 (1.33–1.60)
	No	1,274	1,27,22,945	0.10013	1 (reference)	736	1,27,23,997	0.05784	1 (reference)	1,122	1,27,23,795	0.08818	1 (reference)
Non-smoker	Yes	1,715	1,20,74,401	0.14204	1.06 (0.99–1.14)	660	1,20,76,578	0.054651	1.12 (0.99–1.26)	1,411	1,20,75,595	0.11685	1.12 (1.03–1.21)
	No	2,253	3,31,70,536	0.06792	1 (reference)	621	3,31,74,617	0.018719	1 (reference)	1,252	3,31,73,708	0.03774	1 (reference)
Non-smoker and non-drinker	Yes	1,566	18,29,026	0.13670	1.05 (0.97–1.13)	583	1,14,57,413	0.05088	1.08 (0.95–1.22)	1,029	1,14,56,750	0.10291	1.17 (1.07–1.28)
	No	2,128	50,63,314	0.06642	1 (reference)	569	3,20,40,541	0.01776	1 (reference)	1,179	3,20,39,969	0.03212	1 (reference)
Non-smoker and drinker	Yes	149	98,255	0.24240	1.25 (0.97–1.62)	77	6,14,823	0.12524	1.41 (0.97–2.04)	232	6,14,517	0.37753	0.88 (0.73–1.07)
	No	125	1,78,979	0.11058	1 (reference)	52	11,30,579	0.04599	1 (reference)	223	11,30,250	0.19730	1 (reference)
Smoker and non-drinker	Yes	652	4,49,808	0.23455	1.28 (1.15–1.42)	486	27,80,034	0.17482	1.35 (1.19–1.54)	691	27,79,979	0.24856	1.50 (1.34–1.67)
	No	1,035	17,30,941	0.09505	1 (reference)	572	1,08,89,595	0.05253	1 (reference)	811	1,08,89,609	0.07447	1 (reference)
Smoker and drinker	Yes	168	1,05,274	0.25784	1.03 (0.83–1.27)	155	6,51,522	0.23790	1.31 (1.03–1.65)	276	6,51,466	0.42366	1.36 (1.14–1.61)
	No	239	2,91,848	0.13043	1 (reference)	164	18,32,503	0.0895	1 (reference)	311	18,32,320	0.16973	1 (reference)

*Model* Adjusted for age, gender, smoking status, alcohol intake, exercise, body mass index, and diabetes.

We examined the effect of BP categories at baseline on the risk of oral, laryngeal, and esophageal cancers (Table 4). A significant relationship between the risk of oral cancer and hypertension without medication (HR 1.15, 95% CI 1.05–1.26) was observed after adjusting for confounders. We observed an association between risk of laryngeal cancer and hypertension irrespective of medication (HR 1.25, 95% CI 1.09–1.44 for hypertension without medication; HR 1.26, 95% CI 1.12–1.42 for hypertension with medication). Hypertension including prehypertension was particularly associated with an increased risk of esophageal cancer (HR 1.13, 95% CI 1.04–1.23 for prehypertension; HR 1.47, 95% CI 1.33–1.63 for hypertension without medication; HR 1.31, 95% CI 1.20–1.43 for hypertension with medication). The HR per 10-mmHg increment of SBP was 1.03 (95% CI 1.01–1.04) for oral cancer, 1.04 (95% CI 1.01–1.07) for laryngeal cancer, and 1.07 (95% CI 1.06–1.09) for esophageal cancer, respectively. We also identified significant linear associations per 10-mmHg increase in DBP for oral cancers among those with hypertension (HR 1.04, 95% CI 1.01–1.07), laryngeal cancer (HR 1.05. 95% CI 1.01–1.09), and esophageal cancer (HR 1.10, 95% CI 1.07–1.14).

**Table 4:** Multivariable Cox proportional hazard model for incidence of oral, laryngeal, and esophageal cancer according to the blood pressure categories.

Blood pressure categories	Total number	Oral cancer				Laryngeal cancer				Esophageal cancer			
		No of cases	Person-years	Annual incidence rates	Hazard ratio (95% CI)	No of cases	Person-years	Annual incidence rates	Hazard ratio (95% CI)	No of cases	Person-years	Annual incidence rates	Hazard ratio (95% CI)
Normotension	3,373,344	1,400	21,343,268	0.06559	1 (reference)	484	21,345,599	0.022674	1 (reference)	808	21,345,219	0.03785	1 (reference)
Prehypertension	3,891,818	2,127	24,550,213	0.08664	0.99 (0.92–1.06)	873	24,553,015	0.035556	1.04 (0.93–1.17)	1566	24,552,284	0.06378	1.13 (1.04–1.23)
Hypertension without medication	857,819,857,819	738	5,368,896	0.13746	1.15 (1.05–1.26)	355	5,369,624	0.066113	1.25 (1.09–1.44)	710	5,369,221	0.13224	1.47 (1.33–1.63)
Hypertension with medication	1,623,625	1,797	10,138,425	0.17725	1.07 (0.99–1.16)	946	10,140,092	0.093293	1.26 (1.12–1.42)	1668	10,139,377	0.16451	1.31 (1.20–1.43)
Systolic blood pressure					1.00 (1.00–1.01)				1.00 (1.00–1.01)				1.01 (1.01–1.01)
Diastolic blood pressure					1.00 (1.00–1.01)				1.00 (1.00–1.01)				1.01 (1.01–1.01)
Systolic blood pressure (per 10 mmHg)					1.03 (1.01–1.04)				1.04 (1.01–1.07)				1.07 (1.06–1.10)
Diastolic blood pressure (per 10 mmHg)					1.04 (1.01–1.07)				1.05 (1.01–1.09)				1.10 (1.07–1.14)

*Model* Adjusted for age, gender, smoking status, alcohol intake, exercise, body mass index, and diabetes.

### Impact of a combination of hypertension and diabetes on oral, laryngeal, and esophageal cancer incidence

Multivariable analyses revealed that participants with both hypertension and diabetes had the highest HR for oral cancer (HR 1.22; 95% CI 1.11–1.33), laryngeal cancer (HR 1.46; 95% CI 1.22–1.66), and esophageal cancer (HR 1.44; 95% CI 1.31–1.58) (Table 5). Participants with diabetes were also at significantly higher risk of laryngeal cancer (HR 1.20; 95% CI 1.02–1.42) and esophageal cancer (HR 1.19; 95% CI 1.05–1.35). In addition, subsample analysis demonstrated that men with both hypertension and diabetes had 1.23, 1.49, 1.51 times higher risk of oral cancer, laryngeal cancer, and esophageal cancer, respectively. Hypertension in combination with diabetes was found to be a strong risk factor, with a higher risk estimate for each cancer, in people who were ex- or current smokers as well as in people who had never smoked. The smoking group with hypertension and diabetes was at a higher risk of oral cancer (HR 1.33, 95% CI 1.14–1.56), laryngeal cancer (HR 1.68, 95% CI 1.41–2.00), and esophageal cancer (HR 1.54, 95% CI 1.33–1.79) compared to the smoking group without hypertension or diabetes. Specifically, among even those who had never smoked, there was an association between hypertension in combination with diabetes and the risk of oral cancer (HR 1.17, 95% CI 1.04–1.31), laryngeal cancer (HR 1.27, 95% CI 1.06–1.52), and esophageal cancer (HR 1.35, 95% CI 1.20–1.53).The HR for hypertension and diabetes was 1.16 (95% CI 1.03–1.30) for oral cancer, 1.24 (95% CI 1.02–1.50) for laryngeal cancer, and 1.39 (95% CI 1.21–1.59) for esophageal cancer among non-smokers and non-drinkers.

**Table 5:** Multivariable Cox proportional hazard model for incidence of oral, laryngeal, and esophageal cancer according to the condition of diabetes and hypertension.

Variables	Condition of diabetes/hypertension	Total Number	Oral Cancer				Laryngeal cancer				Esophageal cancer			
			No of cases	Person-years	Annual incidence rates	Hazard ratio (95% CI)	No of cases	Person-years	Annual incidence rates	Hazard ratio (95% CI)	No of cases	Person-years	Annual incidence rates	Hazard ratio (95% CI)
All	None	69,07,641	3,207	4,36,62,173	0.07345	1 (reference)	1,197	4,36,67,030	0.02741	1 (reference)	2,099	4,36,66,008	0.04807	1 (reference)
	Diabetes	3,57,521	320	22,31,308	0.14341	1.10 (0.98–1.24)	160	22,31,585	0.07170	1.20 (1.02–1.42)	275	22,31,495	0.12324	1.19 (1.05–1.35)
	Hypertension	20,03,674	1,927	1,25,66,065	0.15335	1.11 (1.04–1.18)	956	1,25,67,990	0.07607	1.23 (1.12–1.34)	1,781	1,25,67,070	0.14172	1.26 (1.18–1.35)
	Hypertension and diabetes	4,77,770	608	29,41,257	0.20671	1.22 (1.11–1.33)	345	29,41,727	0.11728	1.46 (1.29–1.66)	597	29,41,528	0.20296	1.44 (1.31–1.58)
Male	None	37,10,123	2,310	2,34,00,531	0.09872	1 (reference)	1,118	2,34,03,176	0.04777	1 (reference)	1,872	2,34,02,409	0.79990	1 (reference)
	Diabetes	2,32,117	261	14,40,099	0.18124	1.13 (0.99–1.28)	154	14,40,266	0.10692	1.22 (1.03–1.44)	257	14,40,186	0.17845	1.21 (1.07–1.38)
	Hypertension	11,31,687	1,463	70,47,668	0.20759	1.15 (1.07–1.23)	908	70,48,624	0.12882	1.25 (1.14–1.37)	1,642	70,47,825	0.23298	1.31 (1.22–1.40)
	Hypertension and diabetes	2,81,663	469	17,18,412	0.27293	1.23 (1.11–1.36)	331	17,18,617	0.19260	1.49 (1.31–1.69)	564	17,18,441	0.32820	1.51 (1.37–1.67)
Female	None	31,97,518	897	2,02,61,642	0.04427	1 (reference)	79	2,02,63,854	0.00390	1 (reference)	227	2,02,63,598	0.01120	1 (reference)
	Diabetes	1,25,404	59	7,91,209	0.07457	1.05 (0.80–1.37)	6	7,91,318	0.00758	0.99 (0.43–2.28)	18	7,91,308	0.02275	0.98 (0.61–1.60)
	Hypertension	8,71,987	464	55,18,396	0.08408	1.03 (0.91–1.17)	48	55,19,366	0.00870	0.99 (0.67–1.46)	139	55,19,245	0.02519	0.91 (0.72–1.14)
	Hypertension and diabetes	1,96,107	139	12,22,845	0.11367	1.20 (0.99–1.46)	14	12,23,109	0.01145	1.09 (0.59–1.99)	33	12,23,086	0.02698	0.81 (0.55–1.19)
Smoker	None	19,09,354	1,144	1,20,20,464	0.09517	1 (reference)	648	1,20,21,434	0.05390	1 (reference)	1,010	1,20,21,208	0.08402	1 (reference)
	Diabetes	1,13,457	130	7,02,481	0.18506	1.15 (0.96–1.39)	88	7,02,563	0.12526	1.23 (0.98–1.54)	112	7,02,586	0.15941	1.05 (0.87–1.28)
	Hypertension	4,49,000	621	27,87,010	0.22282	1.23 (1.11–1.37)	465	27,87,172	0.16684	1.34 (1.18–1.52)	742	27,87,016	0.26623	1.46 (1.32–1.61)
	Hypertension and diabetes	1,06,105	199	6,45,911	0.30809	1.33 (1.14–1.56)	176	6,45,966	0.27246	1.68 (1.41–1.20)	225	6,45,986	0.34830	1.54 (1.33–1.79)
Non-smoker	None	49,98,287	2,063	3,16,41,709	0.06520	1 (reference)	549	3,16,45,595	0.01735	1 (reference)	1,089	3,16,44,799	0.03441	1 (reference)
	Diabetes	2,44,064	190	15,28,827	0.12428	1.07 (0.92–1.25)	72	15,29,021	0.04709	1.17 (0.91–1.49)	163	15,28,908	0.10661	1.30 (1.10–1.54)
	Hypertension	15,54,674	1,306	97,79,054	0.13355	1.06 (0.98–1.14)	491	97,80,817	0.05020	1.12 (0.98–1.28)	1,039	97,80,054	0.10624	1.13 (1.04–1.24)
	Hypertension and diabetes	3,71,665	409	22,95,346	0.17819	1.17 (1.04–1.31)	169	22,95,760	0.07361	1.27 (1.06–1.52)	372	22,95,541	0.16205	1.35 (1.20–1.53)
Non-smoker and non-drinker	None	48,31,627	1,956	3,05,85,453	0.06395	1 (reference)	503	3,05,89,199	0.01644	1 (reference)	894	3,05,88,699	0.02923	1 (reference)
	Diabetes	2,31,687	172	14,51,175	0.11852	1.04 (0.89–1.22)	66	14,51,342	0.04548	1.17 (0.90–1.51)	135	14,51,270	0.09302	1.35 (1.13–1.62)
	Hypertension	14,77,259	1,191	92,90,578	0.12819	1.04 (0.96–1.12)	433	92,92,220	0.04660	1.09 (0.95–1.24)	878	92,91,654	0.09449	1.21 (1.09–1.33)
	Hypertension and diabetes	3,50,767	375	21,64,812	0.17323	1.16 (1.03–1.30)	150	21,65,192	0.06928	1.24 (1.02–1.50)	301	21,65,095	0.13902	1.39 (1.21–1.59)
Non-smoker and drinker	None	1,66,610	107	10,53,266	0.10159	1 (reference)	46	10,53,390	0.04367	1 (reference)	195	10,53,103	0.18517	1 (reference)
	Diabetes	12,369	18	77,163	0.23327	1.46 (0.88–2.42)	6	77,189	0.07773	1.04 (0.44–2.44)	28	77,146	0.36295	1.00 (0.79–1.62)
	Hypertension	77,378	115	4,85,605	0.23682	1.35 (1.02–1.78)	58	4,85,715	0.11941	1.39 (0.93–2.08)	161	4,85,530	0.33160	0.83 (0.67–1.04)
	Hypertension and diabetes	20,877	34	1,29,073	0.36342	1.29 (0.86–1.93)	19	1,29,107	0.14716	1.52 (0.88–2.65)	71	1,28,986	0.55044	1.21 (0.91–1.60)
Smoker and non-drinker	None	16,36,555	935	1,03,04,131	0.09074	1 (reference)	500	1,03,05,024	0.04852	1 (reference)	735	1,03,04,978	0.07132	1 (reference)
	Diabetes	94,386	100	5,84,526	0.17108	1.13 (0.92–1.39)	72	5,84,571	0.12317	1.33 (1.04–1.70)	76	5,84,630	0.13000	1.00 (0.79–1.27)
	Hypertension	3,64,093	500	22,58,663	0.22137	1.29 (1.15–1.45)	363	22,58,841	0.16070	1..40 (1.21–1.61)	528	22,58,819	0.23375	1.48 (1.32–1.67)
	Hypertension and diabetes	85,715	152	5,21,096	0.29169	1.33 (1.12–1.60)	123	5,21,192	0.23600	1.56 (1.27–1.92)	163	5,21,160	0.31276	1.58 (1.33–1.89)
Smoker and drinker	None	2,72,780	209	17,14,735	0.12188	1 (reference)	148	17,14,800	0.08631	1 (reference)	275	17,14,653	0.16038	1 (reference)
	Diabetes	19,068	30	1,17,665	0.25496	1.25 (0.85–1.84)	16	1,17,702	0.13594	0.92 (0.55–1.54)	36	1,17,666	0.30595	1.17 (0.82–1.65)
	Hypertension	84,891	121	5,27,247	0.22949	1.02 (0.81–1.29)	102	5,27,234	0.19346	1.15 (0.89–1.50)	214	5,27,120	0.40598	1.38 (1.15–1.670
	Hypertension and diabetes	20,383	47	1,24,327	0.37803	1.32 (0.95–1.83)	53	1,24,288	0.42643	2.02 (1.46–2.80)	62	1,24,346	0.49861	1.43 (1.08–1.90)

*Model* Adjusted for age, gender, smoking status, alcohol intake, exercise, and body mass index.

## Discussion

Results of this population study demonstrated the effect of hypertension and MetS on the development of oral, laryngeal, and esophageal cancers in a nationwide setting of nearly a quarter of the adult population in Korea. As far as we know, this study is one of the few that examined a massive and homogeneous nationwide population-based cohort. We demonstrated that hypertension and MetS had a significant linear relationship with the risk of oral, laryngeal, and esophageal cancers after adjusting for confounders such as gender, smoking status, alcohol intake, exercise, body mass index, and diabetes. MetS was associated with 9%, 27%, and 11% higher risk of developing oral, laryngeal, and esophageal cancers, respectively. Hypertension was also associated with risk of oral, laryngeal, and esophageal cancers. Both hypertension and prehypertension were associated with an increased risk of developing esophageal cancer. Although hypertension was most correlated with these three types of cancer, diabetes was also found to increase the risk of UADT cancers. The combined presence of hypertension and diabetes was associated with an increased risk of oral, laryngeal, and esophageal cancer even among nonsmokers.

Previous studies showing an association between hypertension and cancer are rare. This relationship has not yet been accepted by the scientific community due to conflicting results^6,12,13^. Several recent observational studies have reported an association between hypertension and the development of UADT cancers^14–16^. Some reports have linked anti-hypertensive drugs to the development of certain types of cancer. Calcium channel blockers have been correlated with cancer as they can affect cellular replication and apoptosis by interfering with calcium-mediated intracellular mechanisms^17,18^. There are also reports that the use of diuretics is correlated with a high incidence of renal carcinoma and that the use of beta blockers could increase rates of various types of cancer, such as colorectal and breast cancers^19–21^.

Previous studies have produced conflicting results regarding the relationship between antihypertensive agents and cancer incidence. However, the most recent studies have denied an increased risk of cancer in patients taking antihypertensive drugs^14,22,23^. Likewise, while the assertion that hypertension causes cancer is controversial, reports that hypertension can occur as a side effect of chemotherapy tend to be more reputable. Many anti-cancer drugs have been reported to cause arterial hypertension through different mechanisms^6^. Among chemotherapeutic drugs, anti-VEGF (vascular endothelial growth factors) drugs are most frequently involved in a rise in blood pressure levels mainly through decreased nitric oxide synthesis^6^.

Development of oral squamous cell carcinoma is reportedly influenced by numerous factors, including tobacco, alcohol, diet and nutrition, viruses, radiation, ethnicity, familial and genetic predisposition, oral thrush, immunosuppression, use of mouthwash, syphilis, dental factors, occupational risks, and mate tea^24^. Risk factors for the pathogenesis of laryngeal cancer are tobacco and alcohol consumption, red meat, and exposure to environmental factors such as asbestos, polycyclic aromatic hydrocarbons, and textile dus^25^. The relationship between gastroesophageal reflux disease (GERD) and human papilloma virus in the development of laryngeal cancer is still controversial and under investigation^26,27^. Risk factors for esophageal cancer are known to include alcohol, smoking, tea, mate tea, and coffee^2^. Those for esophageal adenocarcinoma include GERD, Barrett’s esophagitis, obesity, and smoking tobacco^2,28^.

Although this study grouped oral, laryngeal, and esophageal cancers anatomically into the category of UADT cancer, these three cancers have distinct cell types. They are somewhat heterogenous histopathologically. Most oral and laryngeal cancers are squamous cell carcinoma, for which alcohol and smoking are risk factors^1,12,25^. However, the majority of esophageal cancers are squamous cell carcinoma. Adenocarcinomas make up a significant proportion. They are especially predominant among white men^2^. Studies have shown that one of the major risk factors for developing esophageal adenocarcinoma is GERD^28^. Studies have also reported that GERD is more common in patients with hypertension^29^. Drahos et al. reported that MetS is associated with an increased risk of esophageal adenocarcinoma in males without GERD and females regardless of GERD status using a SEER-Medicare-linked database^30^. Thus, one can infer a relationship between hypertension and esophageal adenocarcinoma. However, this relationship has not yet been reported by the scientific community.

Our study has many strengths. First, participants comprised one-fourth of the adult Korean population. In addition, this longitudinal study followed participants for seven years, gathering data on cancer development. Second, the reliability of the data is strengthened by the accessibility of Korea’s healthcare system, which covers all Korean citizens without exception. Third, study data only included cases of cancer that were diagnosed by a pathologist and types of hypertension were classified according to strict diagnostic criteria. Finally, BP was measured by skilled personnel and was not reported on its own. Detailed information on lifestyle, BMI, and laboratory test was also provided, enabling adjustments to potential confounding and shared risk factors.

There were some limitations. First, data on UADT cancers classified by their ICD code did not include information on cancer cell types. As a result, we were unable to separately analyze cases of squamous cell carcinoma and adenocarcinoma. Second, we did not include detailed biochemical information about cancer stage, laryngoscopy findings, family history, or medication history. Third, the incidence of UADT cancers tended to peak between the ages of 60 and 70, but participants in our study were relatively young. Furthermore, mean age of group without hypertension is significantly younger than group with hypertension. For age difference of the two groups, we already statistically adjusted the effect of age. However, for the average age of whole study participants, it is absolutely shortcoming at a population-based study. Notwithstanding these limitations, it would not change the lesson of this study that patients diagnosed with hypertension, even at middle age, should give attention to the risk of UADT cancer. Fourth, our study was subject to the inherent limitations of its retrospective and observational design. We also thought that the follow-up time of this study was rather short to assess the risk of cancer development with improved accuracy. Lastly, we could not evaluate the entire Korean population and therefore our results may have been influenced by selection bias.

In conclusion, this population-based study shows evidence of an association between hypertension/MetS and development of oral, laryngeal, and esophageal cancers. Further research on this subject may lead to recommendations that patients diagnosed with hypertension and MetS should undergo regular screenings for these cancers.

## Materials and methods

### Study population

The Korean National Health Insurance Service (KNHIS), the country’s public medical insurance system, is administered by the Ministry for Health, Welfare and Family Affairs^29^. Korean adults over 40 years of age and employees over 20 years of age receive regular health examinations every one or two years. Detailed information on this program is available in a previous paper^30^. Diagnoses were confirmed using the International Classification of Disease, Tenth Revision, Clinical Modification (ICD-10-CM) codes. Oral, laryngeal, and esophageal cancers were defined as C00–C06, C32.0–32.9, and C15.0–C15.9, respectively. Written informed consent was provided by all participants. The study protocol was approved by the Institutional Review Board of the Catholic Medical Center. Methods were performed in accordance with relevant guidelines and regulations.

### Patient selection

**Figure 2:**
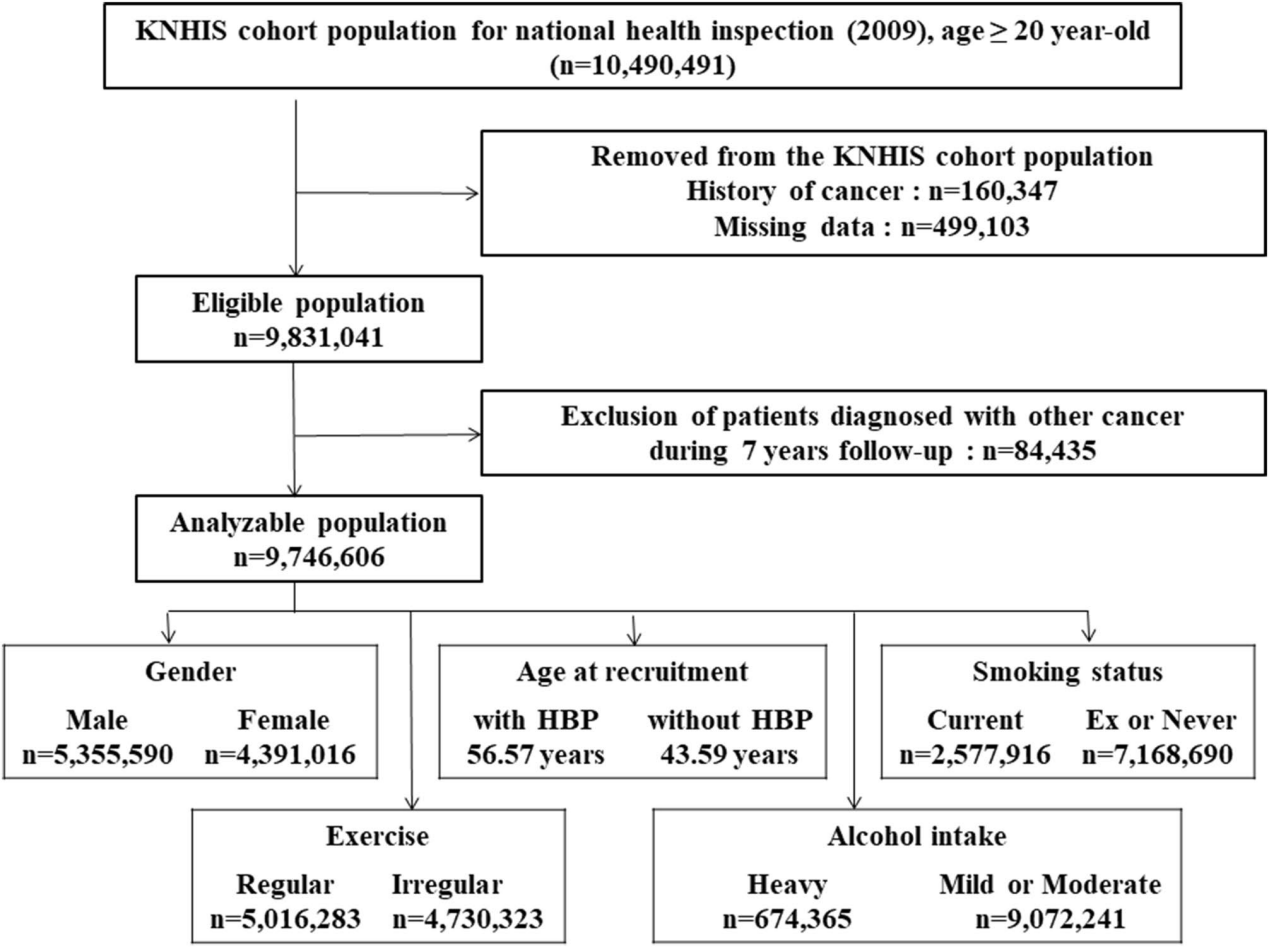
Study profile.

The development of oral, laryngeal, or esophageal cancer was assessed until 2016 using KNHIS claims records during the study period. Basic registration was conducted for participants who had been examined between January 1 and December 31, 2009 (n = 9,746,606) (Figure 2).

Blood pressure (BP) measurements were taken according to the Korean Society of Hypertension guidelines^31^. Briefly, after anthropometric measurements with at least 5 min rest, BP was measured more than twice using a mercury or automatic sphygmomanometer with the patient in a seated position. The measured BP was classified as normal SBP < 120 mmHg and DBP < 80 mmHg), prehypertension (SBP 120–139 mmHg or DBP 80–89 mmHg), or hypertension (SBP ≥ 140 mmHg or DBP ≥ 90 mmHg)^5^. A positive antihypertensive medication history was defined as answering “Yes” to corresponding questions on the health screening questionnaire. The study population was classified into the following four subgroups based on blood pressure and antihypertensive medication history: 1. Normotensive without medication; 2. prehypertensive without medication; 3. hypertensive without medication; and 4. hypertensive with medication. Diabetes was defined as a fasting blood glucose level ≥ 7 mmol/L (≥ 126 mg/dL) or the presence of one or more claims per year for antihyperglycemic medications with ICD-10-CM code E10-14. Dyslipidemia was defined as a total cholesterol level ≥ 6.21 mmol/L (≥ 240 mg/dL) or the presence of one or more claims per year for antihyperlipidemic medications with ICD-10-CM code E78. Body mass index was calculated as weight in kilograms divided by the square of height in meters (kg/m^2^). Medical examinations included measurements of height, weight, and blood pressure, and laboratory tests. Health-related behaviors and past medical history such as smoking, alcohol consumption, and physical activity were collected using standardized self-reporting questionnaires.

The definition of MetS was based on the definition established by the joint interim statement of the International Diabetes Federation Task Force on Epidemiology and Prevention^30^. According to this institution, patients with MetS should have three or more of the following five components: abdominal obesity (≥ 90 cm for men and ≥ 85 cm for women), elevated blood pressure (systolic ≥ 130 and/or diastolic ≥ 85 mmHg), hyperglycemia (fasting plasma glucose ≥ 5.6 mmol/L (≥ 100 mg/dL)), hypertriglyceridemia (triglycerides ≥ 1.7 mmol/L (≥ 150 mg/dL)), and low HDL-cholesterol levels (1.0 mmol/L (< 40 mg/dL) for men and 1.3 mmol/L (< 50 mg/dL) for women)^32^.

### Statistical analysis

All statistical analyses were conducted using SAS software (version 9.2; SAS Institute, Cary, NC, USA). Baseline characteristics of study participants according to the presence of hypertension are presented as means ± standard deviations for continuous variables and numbers (percentages) for categorical variables. Values were compared using independent t-tests for continuous variables and chi-squared tests for categorical variables. Incidence rates of oral, laryngeal, esophageal cancers were calculated by dividing the number of events by 1,000 person-years. Cox proportional hazards analyses were performed to evaluate the association of MetS and hypertension with incidence of oral, laryngeal, or esophageal cancer. HRs and 95% CIs were calculated. Models were adjusted for smoking status, alcohol intake, exercise, body mass index, and diabetes. Kaplan–Meier curves were used to show the cumulative incidence probability of oral, laryngeal, or esophageal cancer. Log-rank tests were performed to examine the association of MetS and hypertension with the risk of oral, laryngeal, or esophageal cancer. A p-value < 0.05 was considered statistically significant.
